# Pain and cognitive function in Korean older adults aged 60 years or more: A retrospective longitudinal study

**DOI:** 10.1097/MD.0000000000039952

**Published:** 2024-10-04

**Authors:** Yun-A Kim, Yoon Jeong Cho, Sang Gyu Kwak, Hae-Jin Ko

**Affiliations:** a Department of Family Medicine, Daegu Catholic University School of Medicine, Daegu, Korea; b Department of Medical Statistics, Daegu Catholic University School of Medicine, Daegu, Korea; c Department of Family Medicine, School of Medicine, Kyungpook National University, Kyungpook National University Hospital, Daegu, Korea.

**Keywords:** activities of daily living, cognitive impairment, dementia, pain

## Abstract

To elucidate the relationship between pain and cognitive decline in adults aged ≥60 years using data from the Korean Longitudinal Study of Aging survey. We included 3,287 older adults aged ≥60 years with a Korean Mini-Mental State Examination score ≥24. We assessed the presence of pain and pain interference using self-administered questionnaires. Pain interference was determined based on whether the pain limited the participants’ activities of daily living. According to this assessment, participants were categorized as no pain, low-impact pain, and high-impact pain. Cognitive function was assessed using the Mini-Mental State Examination and classified into 3 groups: normal, cognitive impairment, and suspected dementia. Potential confounding factors, including pain × survey year, were adjusted in the analyses. We also performed subgroup analyses of participants experiencing pain to elucidate the association between pain interference, suspected dementia, and cognitive impairment. A significant difference in the Mini-Mental State Examination scores was observed between individuals with and without pain (*P* < .001). Pain remained negatively associated with the Mini-Mental State Examination score through the first to the eighth wave even after adjusting for confounding factors (β = ‐1.170, 95 % confidence interval (CI): −0.243, −0.097). Compared to the absence of pain, the presence of pain increased the odds of suspected dementia and cognitive impairment by approximately 1.6 and 1.4 times, respectively (odds ratio [OR] = 1.56, 95% CI: 1.26, 1.93; OR = 1.36, 95% CI: 1.20, 1.54). Compared to low-impact pain, high-impact pain increased the odds of suspected dementia and cognitive impairment by approximately 2.1and 1.5 times, respectively (OR = 2.12, 95% CI: 1.76, 2.56; OR = 1.47, 95% CI: 1.31, 1.65). Pain was negatively associated with Mini-Mental State Examination scores in Korean older adults aged ≥60 years and increased the odds of suspected dementia and cognitive impairment. Furthermore, individuals with high-impact pain exhibited higher risks of both suspected dementia and cognitive impairment than those with low-impact pain.

## 1. Introduction

Pain is the most common cause of hospital visits among older adults,^[1]^ and chronic pain prevalence increases with age.^[2]^ A previous study involving 7000 older adults in a community revealed that more than 50% of the study population experienced pain regularly.^[3]^ Additionally, approximately 82% of older adults aged ≥65 years experience pain, and the pain area increases with age.^[4]^ Chronic pain among older adults is mainly secondary to other medical causes, and musculoskeletal pain is the most common.^[5]^ This suggests a more rapid increase in the use of prescription drugs for pain management among older adults compared with other age groups over the past 20 years.^[6]^

Pain in older adults is associated with limitations in movement and social activities, depression, sleep disorders, and increased use of medical facilities and medical expenses.^[7]^ Additionally, chronic pain affects the cognitive function of older adults.^[8]^ A study examining the association between musculoskeletal pain and cognitive function discovered that these patients had lower memory than those without pain.^[9]^

Patients with widespread pain have a 43% higher risk of dementia and a 29% higher risk of stroke than those without widespread pain.^[10]^ Furthermore, a 16-year retrospective cohort study examining the effects of persistent pain on cognitive decline in approximately 10,000 Caucasian older adults aged ≥60 discovered that memory decline progressed 9.2% faster in those who complained of persistent pain than those who did not.^[11]^ However, these studies were conducted on Caucasians, posing a challenge regarding the generalizability of the results to other races. Besides, results on whether pain increases the risk of cognitive decline are conflicting.^[11–14]^ A previous study has examined the risk of dementia in specific diseases characterized by pain in a Korean population; however,^[15]^ none has examined the association between overall pain and cognitive function in older adults with normal cognitive function.

Therefore, we aimed to examine the changes in cognitive function based on the presence of pain in Korean older adults aged ≥60 years with normal cognitive function.

## 2. Materials and methods

### 2.1. Participants

We used data from the first to the eighth wave of the Korean Longitudinal Study of Aging (KLoSA) survey, conducted biannually since 2006, to study the aging process in Korea. The follow-up survey was completed in 2020. Participants were selected by stratifying middle-aged and older adults aged ≥45 years based on region and residential type, excluding Jeju Island. The survey included information on demographic background, family, health, employment, household income, subjective expectations, and life satisfaction.^[16]^ Since KLoSA conducted biannually, in this study, wave 1 corresponds to 0 years, wave 2 corresponds to 2 years, wave 3 corresponds to 4 years, and so forth.

**Figure 1.**
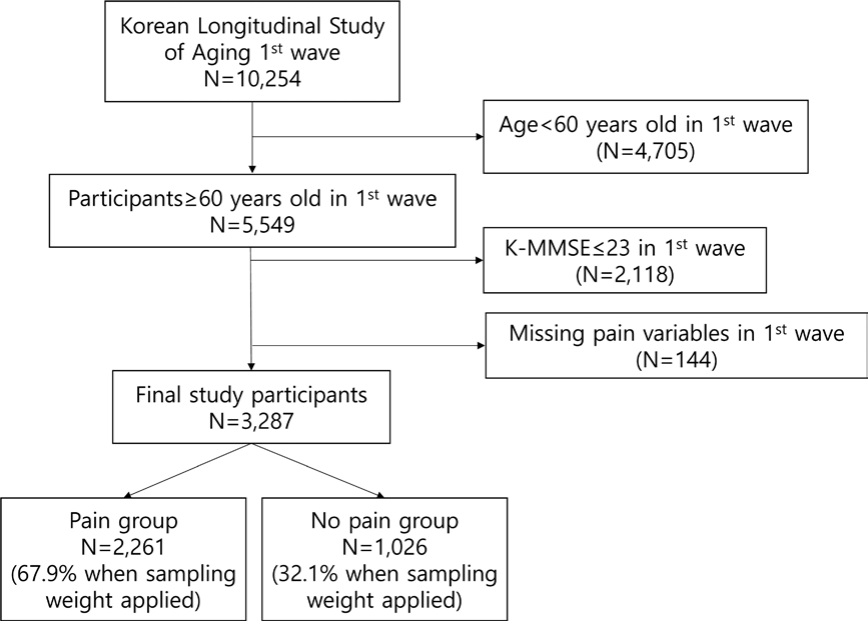
Flow chart of study participants’ selection.

Of the 10,254 individuals who participated in the first wave survey in 2006, we targeted older adults aged ≥60 years with normal cognitive function at the time of the first wave. Normal cognitive function was defined as Korean Mini-Mental State Examination (K-MMSE) score ≥ 24. We included those who completed K-MMSE and responded pain questions in 1st wave. Finally, a total of 3287 participants were selected for analyses (Fig. 1).

We also calculated the missing proportion for each variable to examine the contributed proportion by each participant during the 8th survey in this study (Table S1, Supplemental Digital Content, http://links.lww.com/MD/N674). For age, sex, education level, marital status, employment status, smoking status, alcohol consumption, and regular exercise, the mean missing proportion was 0.26 (standard deviation [SD] = 0.32). For the K-MMSE score, the mean missing proportion was 0.28 (SD = 0.32). No missing data was observed for comorbidities. We also analyzed these missing proportions by each variable and study time using R project (Fig. S1, Supplemental Digital Content, http://links.lww.com/MD/N673).

### 2.2. Study variables

#### 2.2.1. Pain

Pain was assessed using the health status questionnaires of the KLoSA. Participants who responded “no pain” to the question, “In which of the following areas do you feel pain? Please tell us where you are currently experiencing pain,” were classified as the group without pain. Participants who selected one or more options, including head (headache), shoulder, arm, wrist, finger, chest, abdomen, waist, hip, leg, knee, ankle, and toe, were classified as the group with pain. Pain interference was evaluated as pain-related functional limitations. We assessed the participants based on their responses (yes or no) to the question, “Do you find it difficult to perform your daily activities because of this pain?” Among participants with pain, those with activities of daily living (ADL) limitations were considered to have severe pain which referred to high-impact pain, whereas those without ADL limitations were considered to have relatively less severe pain which referred to low-impact pain. In this manner, the presence of pain and pain interference was periodically assessed at every survey.

#### 2.2.2. Cognitive function

Cognitive function was evaluated using the K-MMSE, a screening tool for dementia, which comprises orientation to time and place, memory registration and recall, attention and calculation ability, language ability, and visual construction. The cognitive function is evaluated as a total score of the scores for each question, with 0 and 1 point for incorrect and correct answers, respectively. The total score ranges from 0 to 30, with higher scores indicating better cognitive function.^[17,18]^ K-MMSE was periodically assessed at every survey. We analyzed K-MMSE as continuous score for cognitive function assessment through 1 to 8 waves, and subsequently categorized it as follows; K-MMSE ≤ 17 was classified as suspected dementia, ≥18 or ≤23 as cognitive impairment, and ≥24 as normal.^[16]^

#### 2.2.3. Covariates

Demographic and health status variables were periodically assessed at every survey. Education level was categorized as “elementary school graduates or less,” “junior high school graduates,” “senior high school graduates,” and “college graduates or more.” Marital status was classified as married, previously married but currently without a spouse (such as widowed, separated, or divorced), or single. In addition, employment status was determined using the question, “Are you currently working for income?” Smoking status was categorized into “ever smoker” for those who currently smoke or have smoked ≥100 cigarettes in their lifetime and “never smoker” for those who have smoked <100 cigarettes during their lifetime. Alcohol consumption status was categorized into “ever drinker” and “never drinker” based on the question, “Do you drink alcohol occasionally or often?” Physical activity was evaluated using the question, “Do you exercise at least once a week?” Regarding comorbidities, the presence of each disease was evaluated based on whether a doctor had diagnosed with hypertension, diabetes or hyperglycemia, cancer, heart disease, cerebrovascular disease (CVD), psychiatric problem, and arthritis or rheumatoid disease. Cancer refers to cancers excluding mild skin cancer. Heart diseases include angina, myocardial infarction, heart failure, and other heart diseases. CVD refers to stroke, cerebral hemorrhage, and cerebral infarction. Psychiatric problems include mental disorders such as depression, anxiety, insomnia, excessive stress, and interpersonal difficulties.

### 2.3. Statistical analysis

As the KLoSA data were obtained by stratifying the population age ≥45 years based on region and residential type, a weighted complex sample analysis was performed to estimate the population. A chi-square test for categorical variables and a one-way analysis of variance for continuous variables were performed to compare the baseline characteristics of participants based on the presence of pain. Moreover, a chi-square test was performed to examine changes in the presence of pain and pain interference based on the survey year, and a simple linear regression analysis was performed to examine changes in the K-MMSE value during the survey period.

A linear mixed model (LMM) was used to identify changes in K-MMSE values based on the presence of pain (fixed effect) and the survey year (random effect) which spans to eight waves. A general estimating equation model was conducted to evaluate the risk of suspected dementia and cognitive impairment based on the presence of pain and pain interference through the first to the eighth wave. The survey year, age at the first wave, sex, education level, marital status, employment status, smoking, alcohol consumption, exercise, and comorbidities were adjusted to determine the effect of pain on cognitive functions. Interactions between pain and survey year, as well as between pain interference and survey year, were also adjusted for when performing the LMM and general estimating equation model. The odds ratios (ORs) for suspected dementia and cognitive impairment based on the presence of pain and pain interference were obtained using the cognitive function classification based on K-MMSE values and ADL limitation due to pain. All statistical analyses were performed using SPSS version 21.0 (SPSS Inc., Chicago, IL), and a *P*-value < .05 was considered statistically significant.

## 3. Results

### 3.1. Participants

Among 3287 study participants, 2261 (weighted fraction: 67.9%) and 1026 (weighted fraction: 32.1%) were classified as the groups with and without pain, respectively (Fig. 1). Table 1 presents the characteristics of study participants based on the presence of pain. The mean age of the group with pain was approximately 67.6 years, which was higher than that of the group without pain. Men (77.3%) predominated the group without pain, whereas women (57.5%) predominated the group with pain.

**Table 1:** Baseline characteristics of study participants in the first wave (N*=3287).

		No pain (N = 1026)	Pain (N = 2261)	*P*-value
Age (year)		66.8 ± 0.2	67.6 ± 0.1	<.001
Sex				<.001
	Men (N = 1773)	77.3 (1.4)	42.5 (1.1)	
	Women (N = 1514)	22.7 (1.4)	57.5 (1.1)	
Education level				<.001
	Elementary school or less (N = 1770)	34.6 (1.6)	62.0 (1.1)	
	Junior high school (N = 545)	17.6 (1.3)	16.6 (0.8)	
	Senior high school (N = 670)	30.9 (1.5)	15.9 (0.8)	
	College or more (N = 300)	16.9 (1.3)	5.4 (0.5)	
Marital status				<.001
	Married (N = 2572)	86.2 (1.1)	76.4 (0.9)	
	No longer married or single (N = 715)	13.8 (1.1)	23.6 (0.9)	
Employment status				<.001
	Employed (N = 935)	38.4 (1.6)	26.7 (1.0)	
	Unemployed (N = 2352)	61.6 (1.6)	73.3 (1.0)	
Comorbidities	Hypertension (N = 1197)	29.3 (1.5)	38.4 (1.1)	<.001
	Diabetes or hyperglycemia (N = 504)	12.0 (1.1)	16.3 (0.8)	.002
	Cancer (N = 104)	3.0 (0.6)	3.3 (0.4)	.700
	Heart disease (N = 243)	4.7 (0.7)	8.3 (0.6)	<.001
	Cerebrovascular disease (N = 102)	2.2 (0.5)	3.3 (0.4)	.098
	Psychiatric problem (N = 63)	0.6 (0.2)	2.4 (0.3)	<.001
	Arthritis or rheumatic disease (N = 656)	3.2 (0.5)	27.4 (1.0)	<.001
Smoking				<.001
	Ever smoker (N = 1085)	44.8 (1.7)	27.9 (1.0)	
	Never smoker (N = 2202)	55.2 (1.7)	72.1 (1.0)	
Alcohol drinking				<.001
	Ever drinker (N = 1502)	58.1 (1.6)	40.6 (1.1)	
	Never drinker (N = 1785)	41.9 (1.6)	59.4 (1.1)	
Regular exercise (N = 1452)		55.4 (1.7)	39.4 (1.1)	<.001
K-MMSE score		27.9 ± 0.1	27.2 ± 0.0	<.001

Data are shown as mean ± standard error or proportion (standard error).

K-MMSE = Korean mini-mental status examination.

* The number of participants in each case represents the unweighted value.

A significant difference in education level was observed between the 2 groups. In the group with pain, 62.0% had an elementary school education or less, whereas 5.4% had a college education or more. The respective values in the group without pain were 34.6% and 16.9%. In the group with pain, 76.4% were married. The participants that are currently working are fewer in the group with pain than in the group without pain. A significant difference was observed between the 2 groups based on pain in the prevalence of hypertension, diabetes or hyperglycemia, heart disease, psychiatric problems, and arthritis or rheumatic disease. However, no significant differences were observed between the 2 groups regarding cancer and CVD. Specifically, 27.4% of the participants in the group with pain reported having arthritis or rheumatic disease, a remarkable difference from the 3.2% in the group without pain. The group with pain had a lower proportion of ever-smokers and ever-drinkers than the group without pain. The group with pain exercised less regularly than the group without pain. The K-MMSE score was 27.9 ± 0.1 and 27.2 ± 0.0 in the group without and with pain, respectively.

When comparing included and excluded study participants in baseline survey, there were significant differences in demographics and health status. In addition to lower K-MMSE score in excluded participants, they tended to be older, less educated, unemployed, and more likely not to smoke or drink alcohol compared to included participants (data not shown).

### 3.2. K-MMSE score

Table 2 presents the results of LMM examining the relationship between the presence of pain and the K-MMSE score from baseline to 16 years. Although pain was not associated with the K-MMSE score at baseline, the K-MMSE score was significantly lower in the pain group across 0 years through 16 years after adjusting for all other covariates (β = ‐0.170, 95 % confidence interval [CI]: −0.243, −0.097). When examining the relationship between the presence of pain interference and the K-MMSE score, the K-MMSE score was significantly lower in the pain interference group across 0 years through 16 years (β = ‐0.315, 95 % CI: −0.392, −0.237) (Table 3).

**Table 2:** Linear mixed model* for changes in K-MMSE score through waves 1 to 8 based on the presence of pain.

	Β (SE)	95% CI
Intercept	35.731 (1.022)	33.728, 37.734
Pain	0.144 (0.135)	‐0.121, 0.408
Survey year	0.031 (0.036)	‐0.040, 0.101
Pain × Survey year	‐0.170 (0.037)	‐0.243, ‐0.097

CI = confidence interval, SE = standard error.

* Adjusted for sex, age at the first wave, education level, marital status, employment status, comorbidities, smoking, alcohol consumption, and physical activity.

**Table 3:** Linear mixed model* for changes in K-MMSE score through waves 1 to 8 based on the presence of pain interference.

	Β (SE)	95% CI
Intercept	37.235 (1.215)	34.853, 39.617
Pain interference	0.156 (0.155)	‐0.149, 0.460
Survey year	‐0.006 (0.033)	‐0.070, 0.058
Pain interference × survey year	‐0.315 (0.040)	‐0.392, ‐0.237

CI = confidence interval, SE = standard error.

* Adjusted for sex, age at the first wave, education level, marital status, employment status, comorbidities, smoking, alcohol consumption, and physical activity.

### 3.3. Suspected dementia and cognitive impairment

Compared with the group without pain, the group with pain exhibited a higher OR of 1.56 (95% CI: 1.26, 1.93) for suspected dementia and 1.36 (95% CI: 1.20, 1.54) for cognitive impairment (Table 4). Table 5 presents the subgroup analysis among participants with pain to evaluate the association between the pain interference and cognitive function. Compared with the group with pain without ADL limitations which was referred to low-impact pain, the group with high-impact pain with ADL limitations exhibited a higher OR of 2.12 (95% CI: 1.76, 2.56) and 1.47 (95% CI: 1.31, 1.65) for suspected dementia and cognitive impairment, respectively.

**Table 4:** General estimated equation model* for suspected dementia and cognitive impairment based on the presence of pain.

	Pain	OR (95% CI)
Suspected dementia†	No pain	1.00
	Pain	1.56 (1.26, 1.93)
Cognitive impairment‡	No pain	1.00
	Pain	1.36 (1.20, 1.54)

CI = confidence interval, OR = odds ratio.

* Adjusted for sex, age at the first wave, education level, marital status, employment status, comorbidities, smoking, alcohol consumption, and physical activity.

† K-MMSE ≤ 17.

‡ 18 ≤ K-MMSE ≤ 23.

**Table 5:** General estimated equation model* for suspected dementia and cognitive impairment based on the pain interference among participants with pain.

	Severity of pain	OR (95% CI)
Suspected dementia†	Pain without ADL limitation	1.00
	Pain with ADL limitation	2.12 (1.76, 2.56)
Cognitive impairment‡	Pain without ADL limitation	1.00
	Pain with ADL limitation	1.47 (1.31, 1.65)

ADL = activities of daily living, CI = confidence interval, OR = odds ratio.

* Adjusted for sex, age at the first wave, education level, marital status, employment status, comorbidities, smoking, alcohol consumption, and physical activity.

† K-MMSE ≤ 17.

‡ 18 ≤ K-MMSE ≤ 23.

## 4. Discussion

The K-MMSE score was significantly lowered in the presence of pain through the first to eighth wave, and the odds for suspected dementia and cognitive impairment increased by 1.6 times and 1.4 times, respectively after adjusting for various confounding factors. Furthermore, compared with low-impact pain without ADL limitation, high-impact pain with ADL limitation increased the odds of suspected dementia and cognitive impairment by 2.1 times and 1.5 times, respectively.

Most previous studies have focused on the relationship of specific diseases such as fibromyalgia or diabetic neuropathy,^[8]^ although the association between cognitive function and chronic low back pain^[19]^ or the severity of overall pain^[14]^ was reported. One study that explored the association between headache and cognitive function in 196 adults discovered that MMSE values significantly declined during headache intervals.^[20]^ Moreover, patients with fibromyalgia exhibited lower memory compared with the control group of the same age and education level and with the group of 20 years older.^[21]^ Similarly, patients with fibromyalgia performed poorly in most cognitive function tests; this cognitive decline was associated with pain rather than depression or anxiety symptoms.^[22]^

Our statistical analysis was conducted using the eighth wave of panel data to investigate the longitudinal association of pain with cognitive function. When adjusting for all other covariates, K-MMSE score was lowered in the group with pain through the first to eighth wave. Similarly, in a French study, older adults aged ≥65 years in a community were followed-up for 15 years to examine the association between pain and cognitive function. This study reported that chronic pain lowered the MMSE score by approximately 0.3 points, although this finding was not statistically significant.^[23]^ This French study classified individuals with moderate to severe pain lasting for >6 months as the chronic pain group, whereas we categorized those with pain at the time of the survey as the pain group. Moreover, in this French study, pain was assessed only during the first survey, making it challenging to identify the changes in pain over time. However, the presence of pain was evaluated during each survey period in our study.

In our study, the group with pain showed approximately 1.6 times higher odds of suspected dementia than the group without pain, and the high-impact pain group with ADL limitations showed approximately 2.1 times higher odds than the low-impact pain group without ADL limitations. In a previous study, the risk of dementia progressed 7.7% more rapidly in older adults with persistent pain than in the control group.^[11]^ Additionally, a follow-up study revealed that the group with widespread chronic pain had a 43% increased risk for dementia than the group without widespread chronic pain.^[10]^ However, the pain area investigated in the KLoSA lacked information on widespread chronic pain, which can only be diagnosed in the presence of axial pain in all areas relative to the waist (upper, lower, right, and left).

The MMSE is widely used as a screening tool for cognitive impairment because it can briefly evaluate cognitive function.^[24–26]^ However, its sensitivity for screening mild cognitive impairment varies from 13% to 97%, and its specificity ranges from 60% to 100%.^[24]^ It cannot distinguish relatively mild memory impairment,^[27]^ making it less suitable as a single test for screening mild cognitive impairment.^[28,29]^ Nonetheless, when examining the odds of cognitive impairment based on the presence of pain and pain interference, the group with pain exhibited a higher odds of cognitive impairment compared to those without pain, and the high-impact pain group with ADL limitations showed a higher odds than those without such limitations, indicating that the likelihood for cognitive impairment increases with pain interference.

In total, 67.9% of our participants reported experiencing pain, and approximately 40% complained of severe pain that interfered with ADL. According to a report based on 3 different surveys conducted with Korean elderly, Korean aged ≥ 60 years reportedly had chronic pain rates of 87.7% and 63.8% in women and men, respectively. In addition, musculoskeletal system was the most common site of chronic pain, with the highest prevalence of pain in the knee and lower back. The distribution of musculoskeletal pain differed according to education level, income, occupation, and residential area.^[30]^ Similar to this report, women complained more pain than men, and significant differences were also observed between groups with and without pain in education level, marital status, and employment status in our study. Besides, musculoskeletal pain was the most common complaint in our study (data not shown). According to previous studies, 25% to 50% of older adults in a community^[31,32]^ and 45% to 80% of those in nursing facilities reported having pain.^[33]^ The prevalence of pain was higher in our study than that reported in previous studies on older adults living in a community. This finding may be because our study evaluated the presence of pain at the survey time, thus including cases of both acute and chronic pain.

Several possible mechanisms have been suggested for how pain affects cognitive function. One hypothesis proposes that brain regions involved in pain processing anatomically overlap with those responsible for cognition, leading to competition between pain and cognitive function.^[8]^ Another hypothesis suggests that pain requires attention and consumes cognitive resources, which may affect memory.^[34]^ Additionally, a study examining brain volume in patients with chronic back pain discovered that the gray matter volume was 5% to 11% smaller in the chronic back pain group than in the control group.^[35]^ Hence, changes in brain structure due to neuroplasticity, in patients with chronic pain may contribute to cognitive decline.^[36]^ In addition, pain can affect cognitive function through changes in neurotransmitters, such as gamma-aminobutyric acid.^[37]^

This study had some limitations. First, the duration or frequency of pain was not investigated, and only the presence of pain was identified during the survey. Thus, acute and chronic pains could not be distinguished. Moreover, the KLoSA did not investigate pain management and the use of specific drugs, such as narcotic analgesics and antihistamines, which affect cognitive decline; hence, these were not evaluated in this study. Besides, potential selection bias may exist as predominantly healthy individuals have health checkup, and those who have health issues can be excluded from regular investigation such as KLoSA. Lastly, this was an observational study; thus, clarifying the causal relationship between pain and cognitive decline was challenging.

We periodically evaluated pain and cognitive function using a 14-year follow-up panel data representing Korean older adults. Compared with previous studies that reported the association between pain and cognitive function, this is the first longitudinal study to elucidate the association between pain and cognitive function in older Korean adults with normal cognitive function. We adjusted for demographic and health variables that could affect cognitive function and the interaction between pain and survey year during statistical analysis. Thus, we could identify the association between pain and cognitive function.

In the future, interventional clinical studies will be required to determine whether well-controlled pain can delay cognitive decline or improve cognitive function. Furthermore, it is recommended that clinicians actively manage the pain commonly observed among older adults.

## Author contributions

**Conceptualization:** Yun-A Kim.

**Data curation:** Yun-A Kim, Yoon Jeong Cho, Sang Gyu Kwak.

**Formal analysis:** Yun-A Kim, Sang Gyu Kwak.

**Methodology:** Yun-A Kim, Yoon Jeong Cho, Sang Gyu Kwak.

**Supervision:** Yoon Jeong Cho.

**Writing – original draft:** Yun-A Kim.

**Writing – review & editing:** Yoon Jeong Cho, Hae-Jin Ko.

## Supplementary Material


