# Supplementary material for: Pain and cognitive function in Korean older adults aged 60 years or more: A retrospective longitudinal study
Source: Medicine (Baltimore). 2024 Oct 4;103(40):e39952. doi: 10.1097/MD.0000000000039952 (PMC11460889; doi:10.1097/MD.0000000000039952)
Supplement: Supplementary file 1 [file medi-103-e39952-s001.docx]

**Supplementary Table.** The proportion of missing values at each survey and, the mean, standard deviation, minimum and maximum of the proportion of missing values for each observation during the 8th survey.

| Variable | Proportion | | | | | | | | Mean | Standard deviation | Minimum | Maximum |
| --- | --- | --- | --- | --- | --- | --- | --- | --- | --- | --- | --- | --- |
|  | 1 | 2 | 3 | 4 | 5 | 6 | 7 | 8 |  |  |  |  |
| Age | 0.00 | 0.13 | 0.19 | 0.24 | 0.29 | 0.34 | 0.40 | 0.45 | 0.26 | 0.32 | 0.00 | 0.88 |
| Sex | 0.00 | 0.13 | 0.19 | 0.24 | 0.29 | 0.34 | 0.40 | 0.45 | 0.26 | 0.32 | 0.00 | 0.88 |
| Education level | 0.00 | 0.13 | 0.19 | 0.24 | 0.29 | 0.34 | 0.40 | 0.45 | 0.26 | 0.32 | 0.00 | 0.88 |
| Marital status | 0.00 | 0.13 | 0.19 | 0.24 | 0.29 | 0.34 | 0.40 | 0.45 | 0.26 | 0.32 | 0.00 | 0.88 |
| Employment status | 0.00 | 0.13 | 0.19 | 0.24 | 0.29 | 0.34 | 0.40 | 0.45 | 0.26 | 0.32 | 0.00 | 0.88 |
| Hypertension | 0.00 | 0.00 | 0.00 | 0.00 | 0.00 | 0.00 | 0.00 | 0.00 | 0.00 | 0.00 | 0.00 | 0.00 |
| Diabetes of hyperglycemia | 0.00 | 0.00 | 0.00 | 0.00 | 0.00 | 0.00 | 0.00 | 0.00 | 0.00 | 0.00 | 0.00 | 0.00 |
| Cancer | 0.00 | 0.00 | 0.00 | 0.00 | 0.00 | 0.00 | 0.00 | 0.00 | 0.00 | 0.00 | 0.00 | 0.00 |
| Cerebrovascular disease | 0.00 | 0.00 | 0.00 | 0.00 | 0.00 | 0.00 | 0.00 | 0.00 | 0.00 | 0.00 | 0.00 | 0.00 |
| Psychiatric problem | 0.00 | 0.00 | 0.00 | 0.00 | 0.00 | 0.00 | 0.00 | 0.00 | 0.00 | 0.00 | 0.00 | 0.00 |
| Arthritis or rheumatoid disease | 0.00 | 0.00 | 0.00 | 0.00 | 0.00 | 0.00 | 0.00 | 0.00 | 0.00 | 0.00 | 0.00 | 0.00 |
| Heart disease | 0.00 | 0.00 | 0.00 | 0.00 | 0.00 | 0.00 | 0.00 | 0.00 | 0.00 | 0.00 | 0.00 | 0.00 |
| Smoking status | 0.00 | 0.13 | 0.19 | 0.24 | 0.29 | 0.34 | 0.40 | 0.45 | 0.26 | 0.32 | 0.00 | 0.88 |
| Alcohol consumption | 0.00 | 0.13 | 0.19 | 0.24 | 0.29 | 0.34 | 0.40 | 0.45 | 0.26 | 0.32 | 0.00 | 0.88 |
| Regular exercise | 0.00 | 0.13 | 0.19 | 0.24 | 0.29 | 0.34 | 0.40 | 0.45 | 0.26 | 0.32 | 0.00 | 0.88 |
| K-MMSE score | 0.00 | 0.16 | 0.23 | 0.27 | 0.32 | 0.37 | 0.43 | 0.49 | 0.28 | 0.32 | 0.00 | 0.88 |
| Total | 0.00 | 0.08 | 0.12 | 0.14 | 0.18 | 0.21 | 0.24 | 0.27 | 0.12 | 0.15 | 0.00 | 0.41 |
