# Supplementary figures and images for: Pain and cognitive function in Korean older adults aged 60 years or more: A retrospective longitudinal study
Source: Medicine (Baltimore). 2024 Oct 4;103(40):e39952. doi: 10.1097/MD.0000000000039952 (PMC11460889; doi:10.1097/MD.0000000000039952)

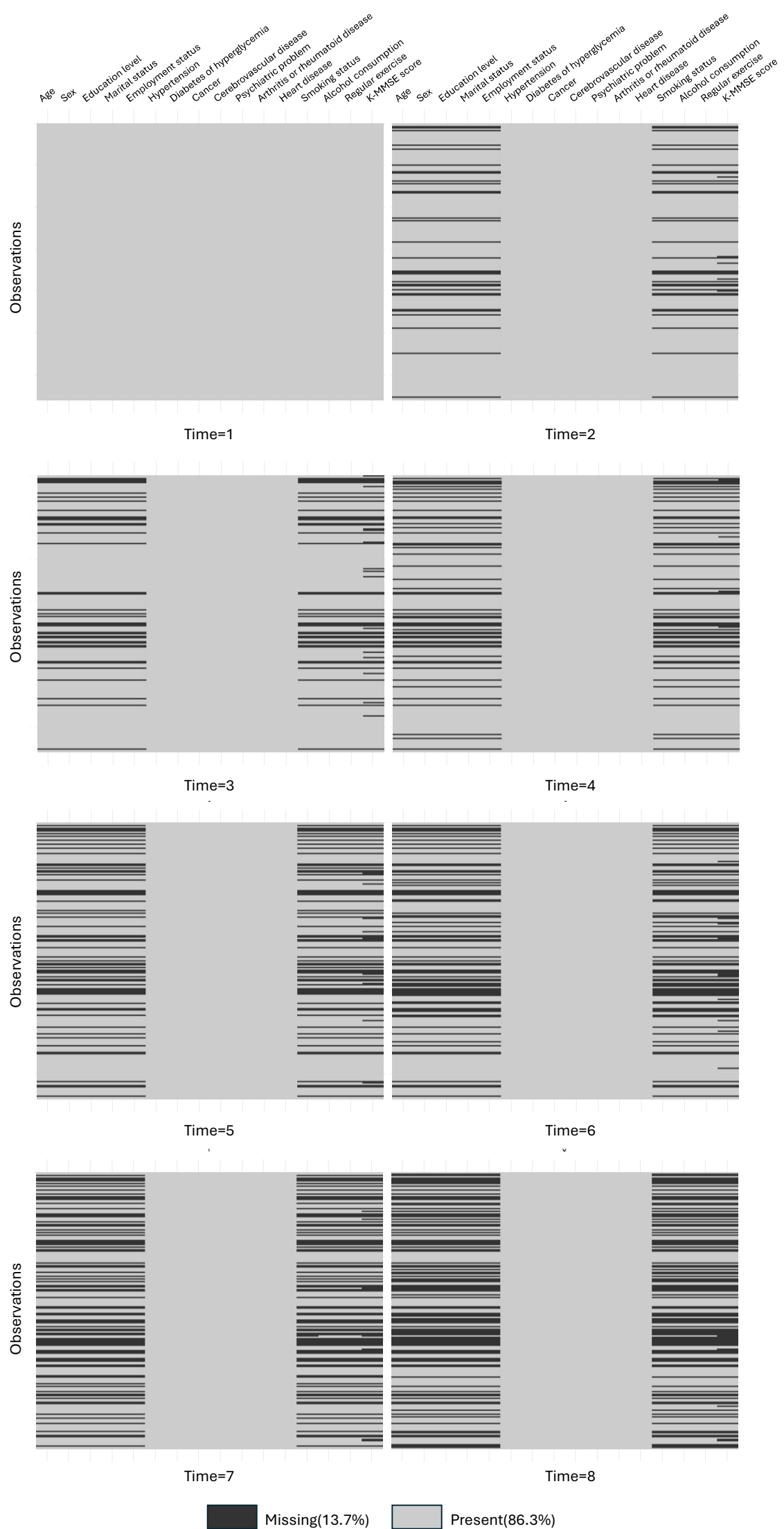

Supplement: Supplementary file 2 [file medi-103-e39952-s002.pdf]
